# Supplementary material for: Transcriptional Reprogramming and Constitutive PD-L1 Expression in Melanoma Are Associated with Dedifferentiation and Activation of Interferon and Tumour Necrosis Factor Signalling Pathways
Source: Cancers (Basel). 2021 Aug 24;13(17):4250. doi: 10.3390/cancers13174250 (PMC8428231; doi:10.3390/cancers13174250)
Supplement: Supplementary file 1 [file cancers-13-04250-s001.zip › cancers-1280223-Table S1.pdf]

Table S1. Mutation and group information of the melanoma cell lines used,

Melanoma cell line	Mutation status	Group
CM138	*BRAF* (V600E)	PD-L1$_{IND}$
CM150.post	*BRAF* (V600E)	PD-L1$_{IND}$
CM145.pre	*BRAF* (V600E)	PD-L1$_{IND}$
CM145.post	*BRAF* (V600E)	PD-L1$_{IND}$
NZM22	WT	PD-L1$_{IND}$
NZM42	*NRAS* (Q61K)	PD-L1$_{IND}$
NZM12	BRAF (V600E)	PD-L1$_{IND}$
NZM15	*NRAS* (Q61K)	PD-L1$_{IND}$
WM115	*BRAF* (V600D)	PD-L1$_{IND}$
WM2664	*BRAF* (V600D)	PD-L1$_{IND}$
CM143.pre	*BRAF* (V600E)	PD-L1$_{CON}$
CM143.post	*BRAF* (V600E)	PD-L1$_{CON}$
NZM9	WT	PD-L1$_{CON}$
NZM40	*NRAS* (Q61H)	PD-L1$_{CON}$
COLO239F	*BRAF* (V600E)	PD-L1$_{CON}$
MM127	*NRAS* (G13R)	PD-L1$_{CON}$
MM595	*BRAF* (V600E)	PD-L1$_{CON}$
