# Supplementary figures and images for: Transcriptional Reprogramming and Constitutive PD-L1 Expression in Melanoma Are Associated with Dedifferentiation and Activation of Interferon and Tumour Necrosis Factor Signalling Pathways
Source: Cancers (Basel). 2021 Aug 24;13(17):4250. doi: 10.3390/cancers13174250 (PMC8428231; doi:10.3390/cancers13174250)

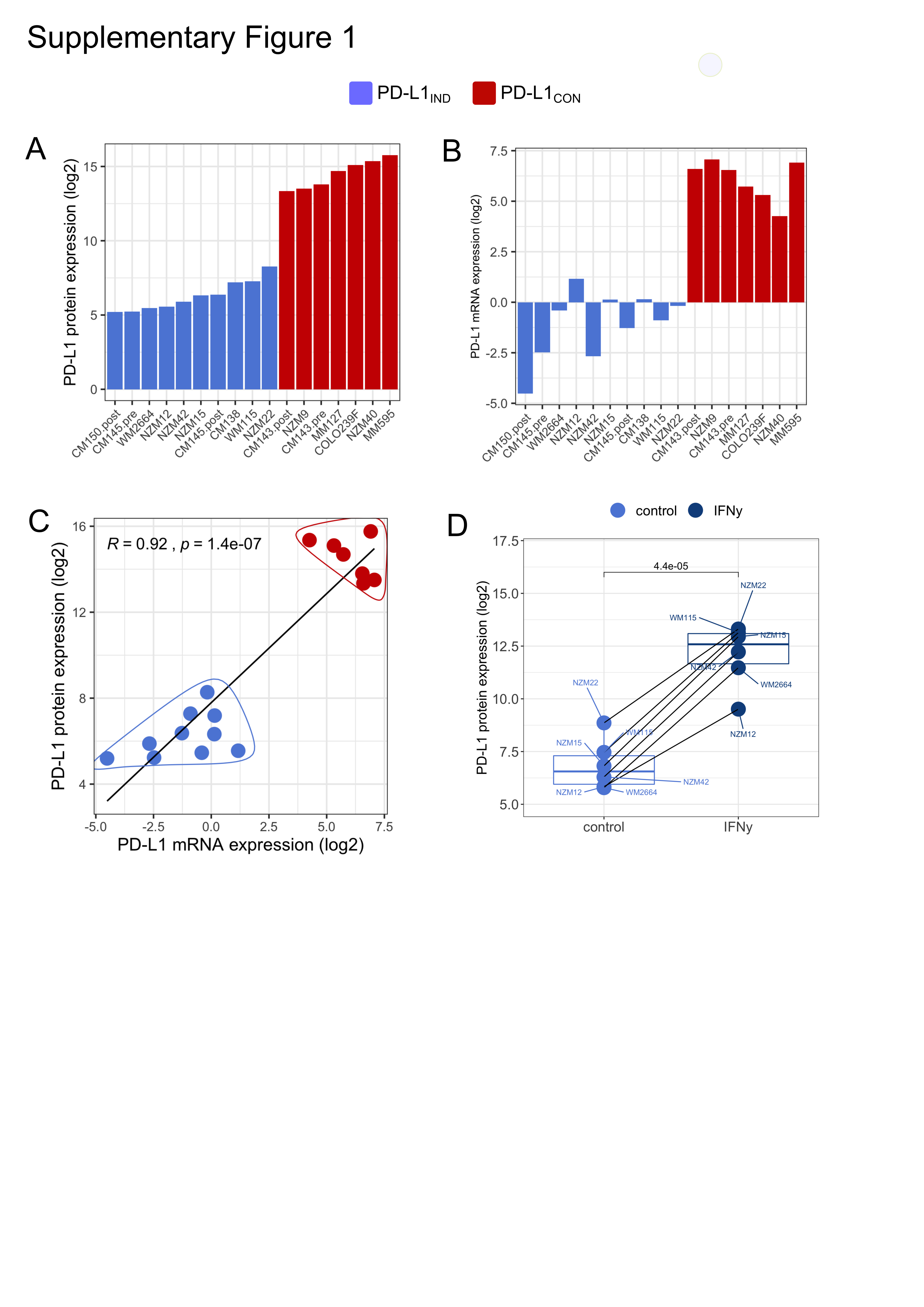

Supplement: Supplementary file 1 [file cancers-13-04250-s001.zip › Supplementary-Figure S1.tif]

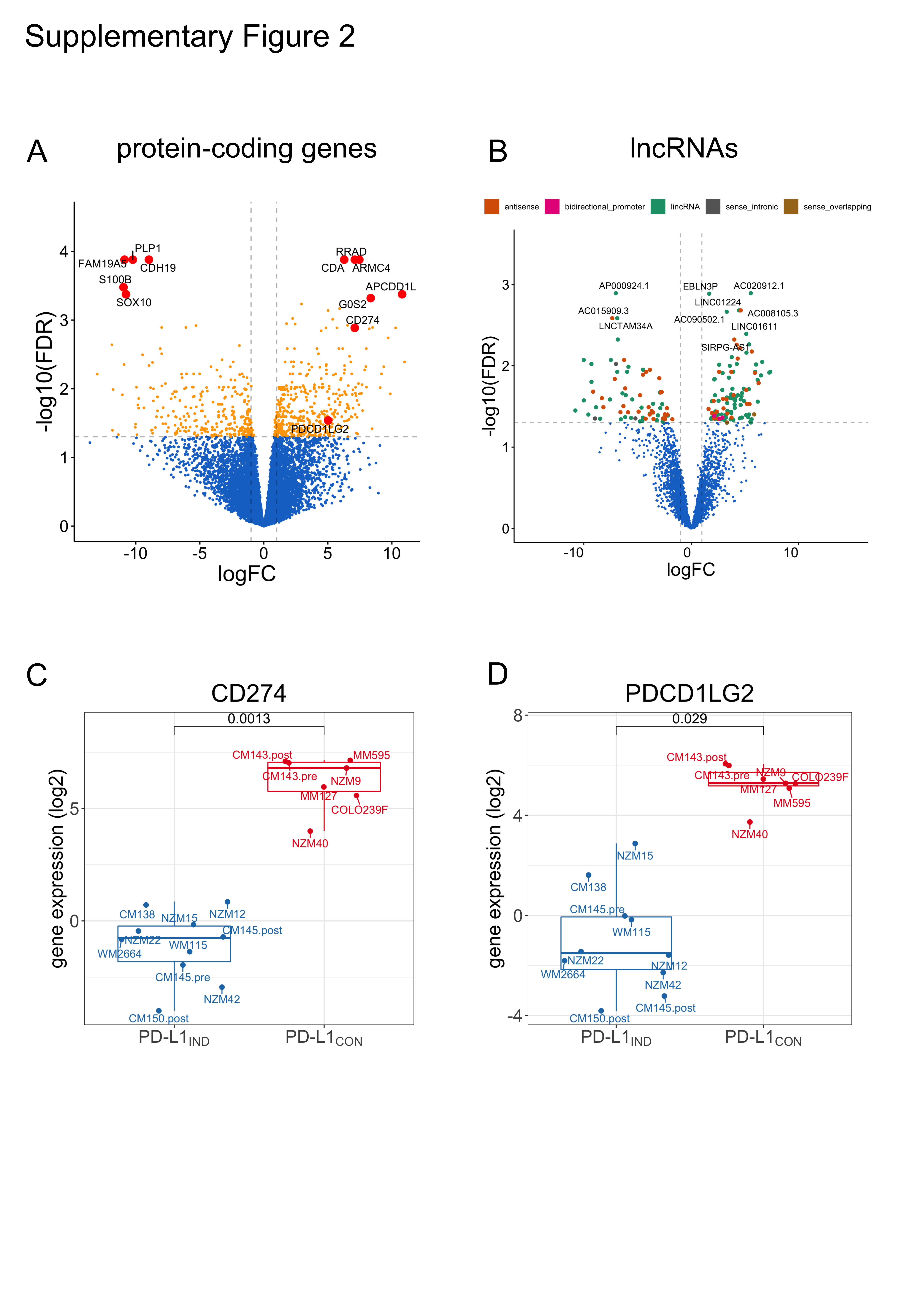

Supplement: Supplementary file 1 [file cancers-13-04250-s001.zip › Supplementary-Figure S2.tif]

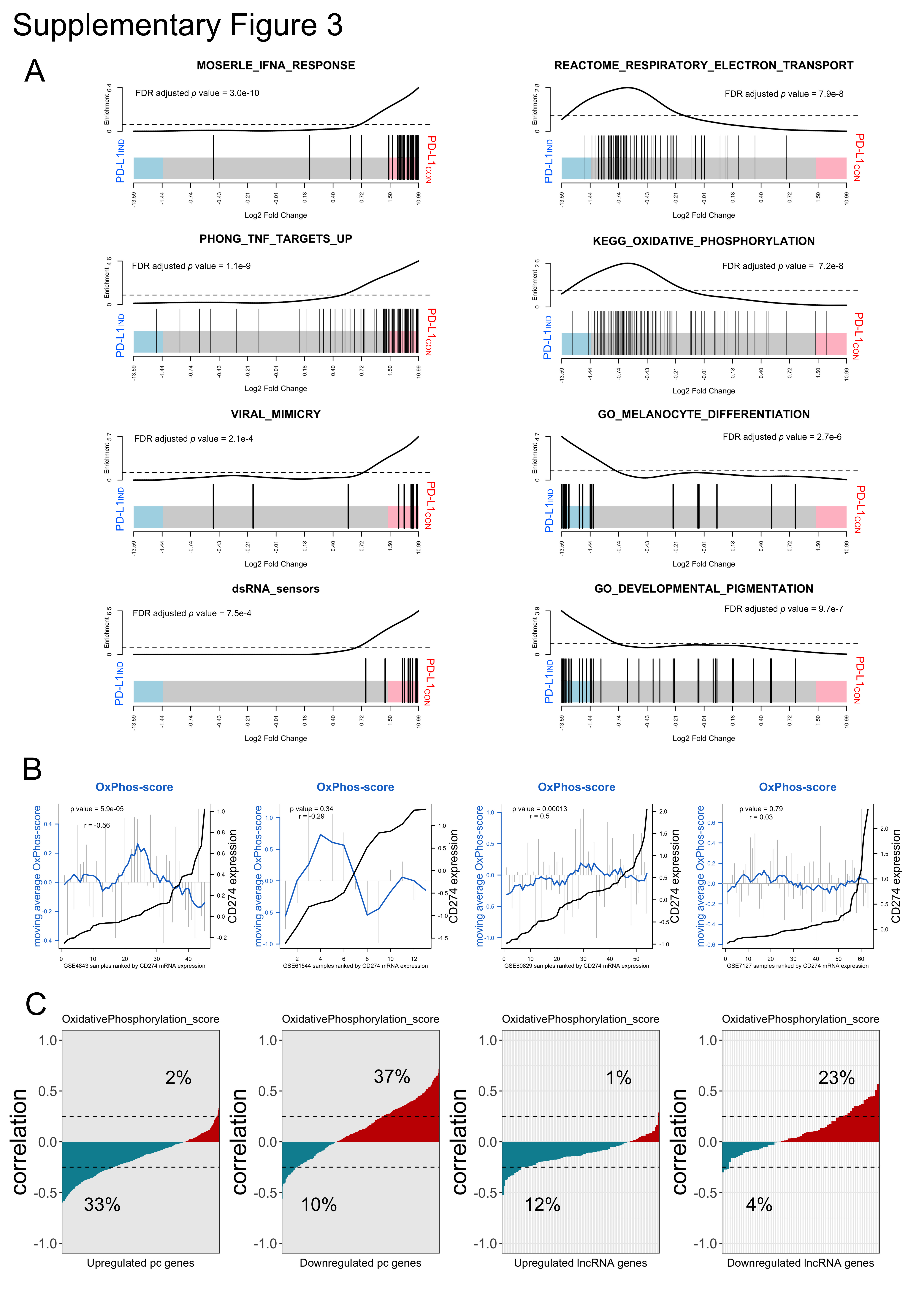

Supplement: Supplementary file 1 [file cancers-13-04250-s001.zip › Supplementary-Figure S3.tif]

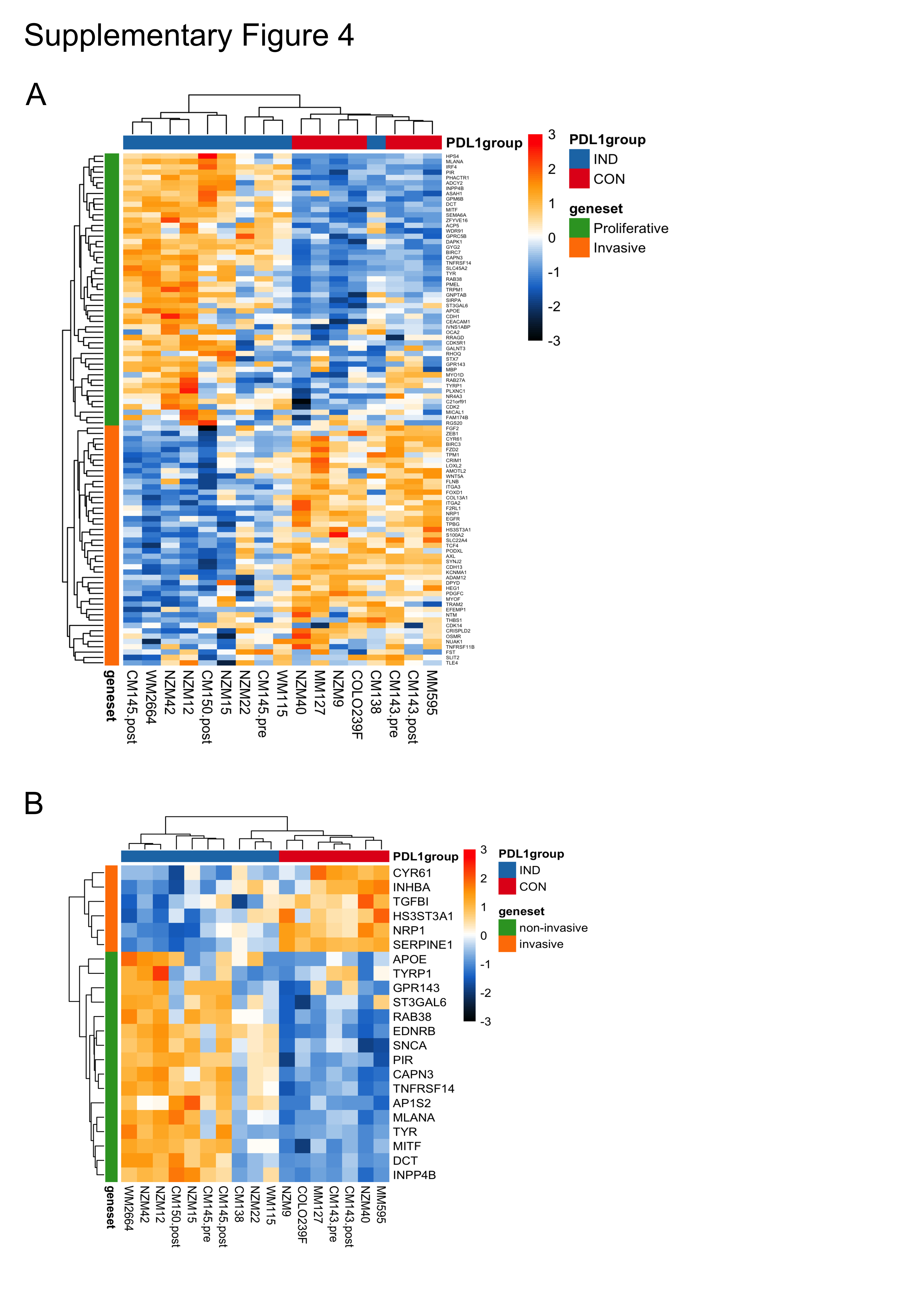

Supplement: Supplementary file 1 [file cancers-13-04250-s001.zip › Supplementary-Figure S4.tif]

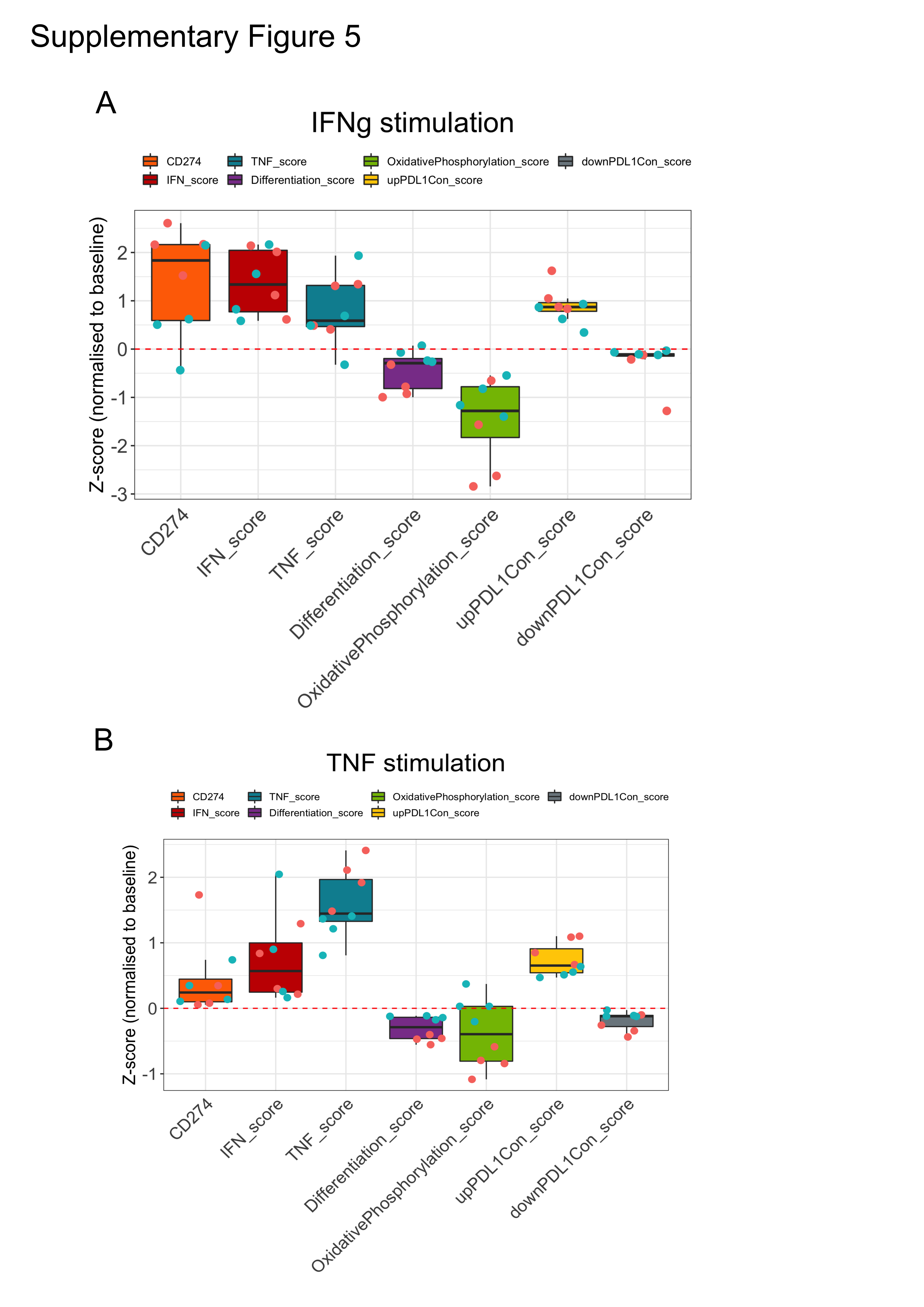

Supplement: Supplementary file 1 [file cancers-13-04250-s001.zip › Supplementary-Figure S5.tif]

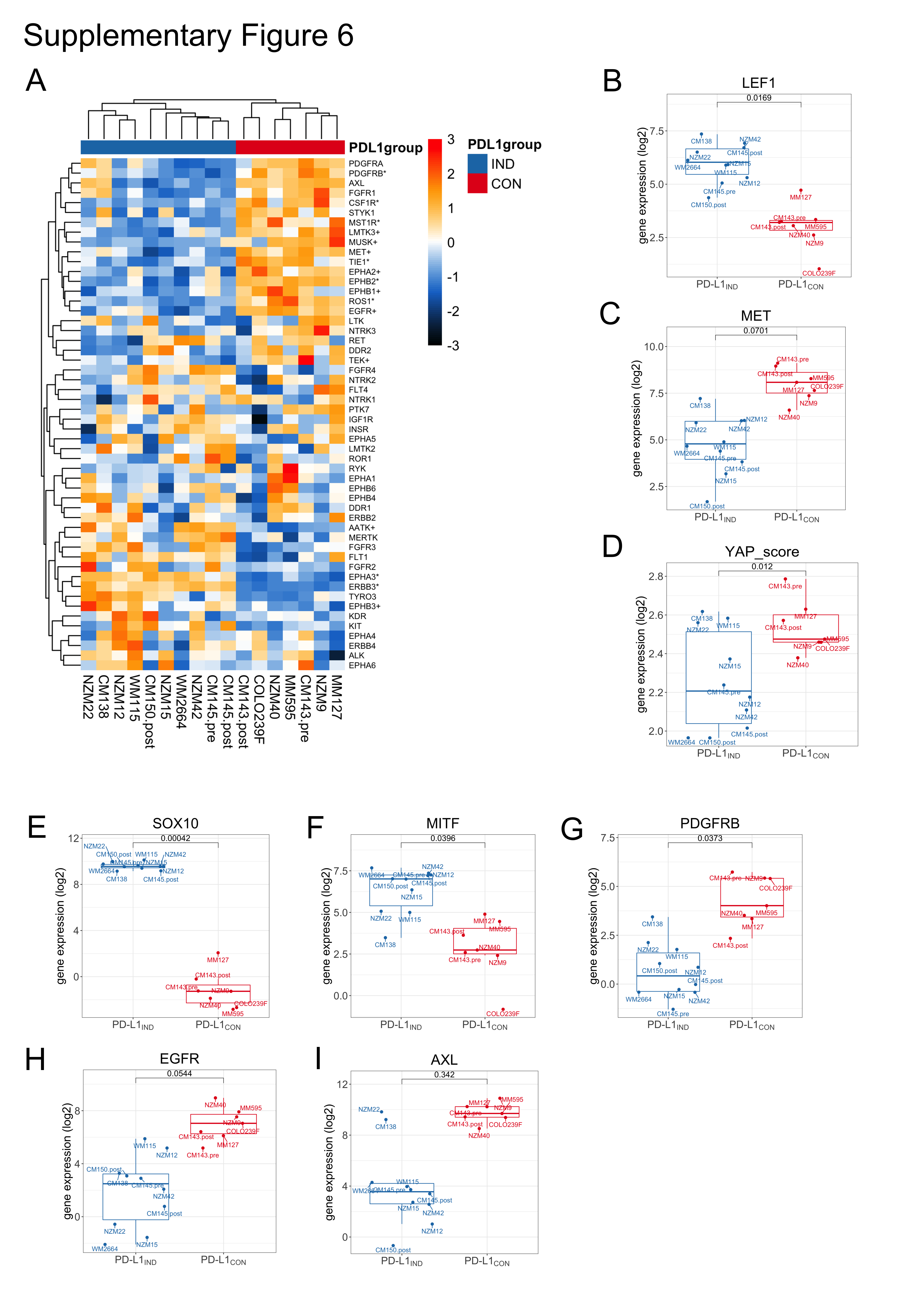

Supplement: Supplementary file 1 [file cancers-13-04250-s001.zip › Supplementary-Figure S6.tif]

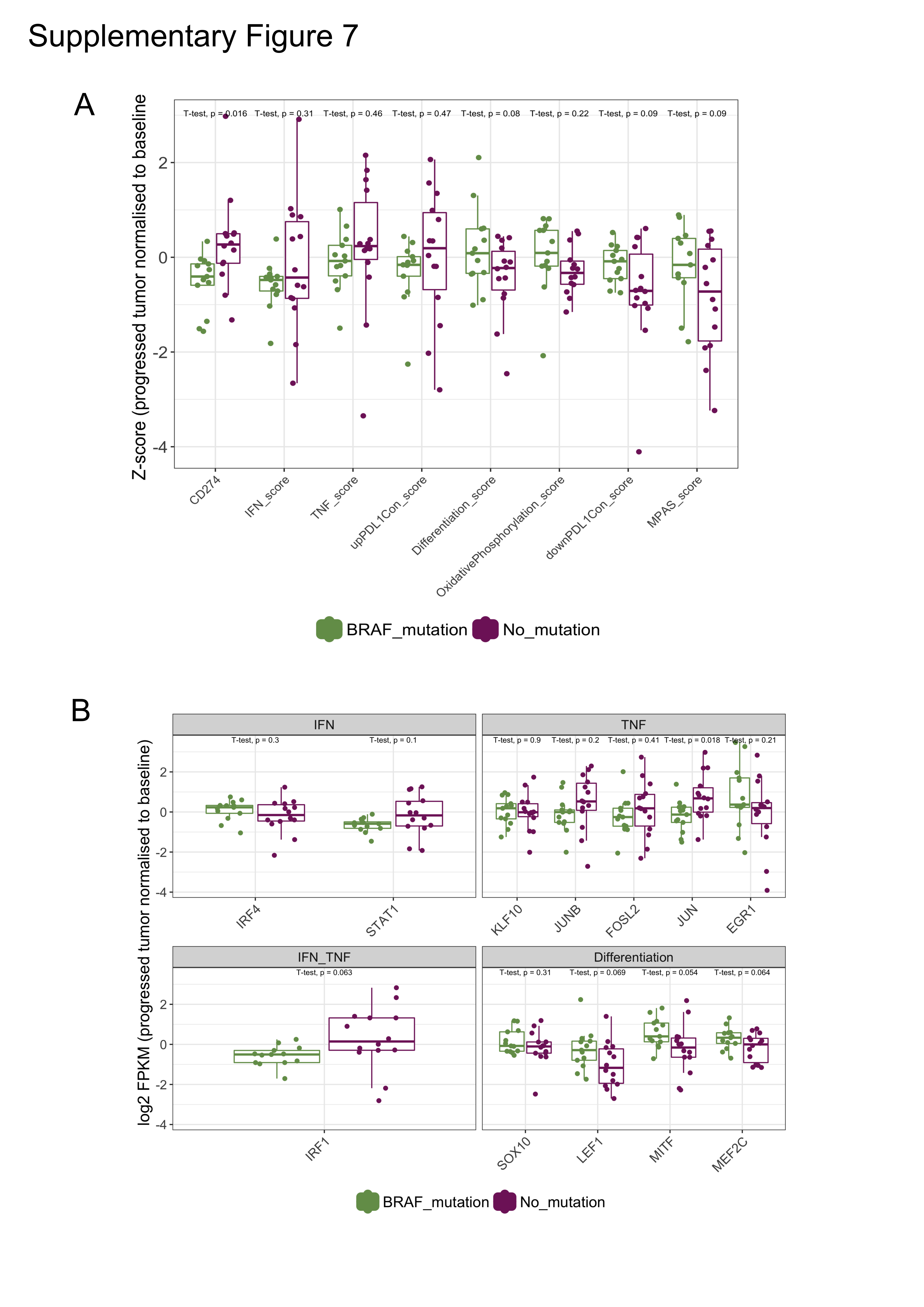

Supplement: Supplementary file 1 [file cancers-13-04250-s001.zip › Supplementary-Figure S7.tif]
